# Supplementary material for: Splice-Junction-Based Mapping of Alternative Isoforms in the Human Proteome
Source: Cell Rep. Author manuscript; Available in PMC 2020 Jan 15. (PMC6961840; doi:10.1016/j.celrep.2019.11.026)
Supplement: 2 [file NIHMS1546469-supplement-2.zip › DF1/PXD010154/Liver/Liver_24_SYPL1_ETSLHSPSNTSAPHSQGGIPPPTGL.pdf]

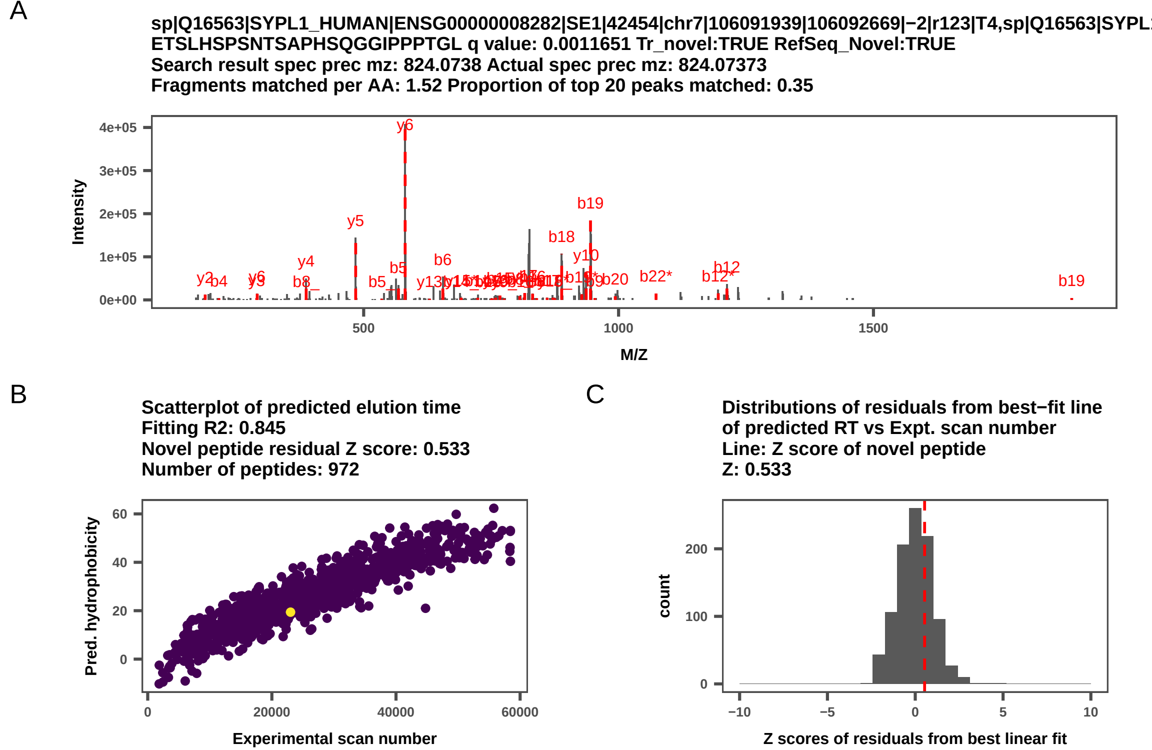
A
sp|Q16563|SYPL1_HUMAN|ENSG00000008282|SE1|42454|chr7|106091939|106092669|−2|r123|T4,sp|Q16563|SYPL
ETSLHSPSNTSAPHSQGGIPPPTGL q value: 0.0011651 Tr_novel:TRUE RefSeq_Novel:TRUE
Search result spec prec mz: 824.0738 Actual spec prec mz: 824.07373
Fragments matched per AA: 1.52 Proportion of top 20 peaks matched: 0.35
Intensity
4e+05
3e+05
2e+05
1e+05
0e+00
y6
y5
b19
b18
y10
b6
b5
y4
y2
b4
y6
y3
b8_
b5_
y13
b20
b22*
b12
b12*
b19
500
1000
1500
M/Z
B
Scatterplot of predicted elution time
Fitting R2: 0.845
Novel peptide residual Z score: 0.533
Number of peptides: 972
Pred. hydrophobicity
60
40
20
0
0
20000
40000
60000
Experimental scan number
C
Distributions of residuals from best−fit line
of predicted RT vs Expt. scan number
Line: Z score of novel peptide
Z: 0.533
count
200
100
0
−10
−5
0
5
10
Z scores of residuals from best linear fit
